# Nur77 deficiency leads to systemic inflammation in elderly mice

**DOI:** 10.1186/s12950-015-0085-0

**Published:** 2015-06-26

**Authors:** Xiu-Ming Li, Xing-Xing Lu, Qian Xu, Jing-Ru Wang, Shen Zhang, Peng-Da Guo, Jian-Ming Li, Hua Wu

**Affiliations:** Pathology Center and Department of Pathology, Soochow University, Suzhou, 215123 China

**Keywords:** Nur77, Inflammation, Immunity, Animal study

## Abstract

**Background:**

Nur77, an orphan member of the nuclear receptor superfamily, has been implicated in the regulation of inflammation. However, the *in vivo* function of Nur77 remains largely unexplored. In the current study, we investigated the role of Nur77 in inflammation and immunity in mice.

**Findings:**

We found that elderly 8-month-old Nur77-deficient mice (Nur77^−/−^) developed systemic inflammation. Compared to wild-type (WT) mice (Nur77^+/+^), Nur77^−/−^ mice showed splenomegaly, severe infiltration of inflammatory cells in several organs including liver, lung, spleen and kidney, increased hyperplasia of fibrous tissue in the lung and enlargement of kidney glomeruli. Additionally, Nur77^−/−^ mice had increased production of pro-inflammatory cytokines and immunoglobulin, and elicited pro-inflammatory M1-like polarization in macrophages as revealed by increased expression of CXCL11 and INDO, and decreased expression of MRC1.

**Conclusions:**

These *in vivo* observations provide evidence for a pivotal role for Nur77 in the regulation of systemic inflammation and emphasize the pathogenic significance of Nur77 *in vivo*.

**Electronic supplementary material:**

The online version of this article (doi:10.1186/s12950-015-0085-0) contains supplementary material, which is available to authorized users.

## Introduction

Nur77 (also called TR3 or NR4A1) belongs to the subfamily 4, group A (NR4A) of nuclear receptor that also comprises Nurr-1 (NR4A2) and NOR-1 (NR4A3) [1]. Accumulating studies have revealed a critical role of Nur77 in the regulation of cancer development [1], metabolism [2], and inflammation [3, 4]. Published findings have indicated that Nur77 is frequently overexpressed in a variety of cancer types, such as colon cancer and pancreatic cancer, and drives cancer development and progression [5, 6]. Nur77 also controls glucose metabolism through regulation of LKB1-AMPK signaling axis [2]. Nur77 is also aberrantly expressed in atherosclerotic lesions [7] and multiple sclerosis [8], indicating that abnormal expression of Nur77 is implicated in the development of immune and inflammatory diseases. Nur77 can be rapidly induced by several inflammatory stimuli such as tumor necrosis factor-alpha (TNFα) and lipopolysaccharide (LPS) in macrophages and monocytes [7, 9]. Moreover, Nur77 can interact with interferon-stimulated gene 12 (ISG12), a cofactor that stimulates nuclear export of nuclear receptors [10] and a critical modulator of innate immune responses in murine models of sepsis [11], further suggesting the potential critical role of Nur77 in the regulation of inflammation and immunity.

However, previous data has revealed that Nur77 plays dual, contradictory roles in inflammation. On the one hand, Nur77 may mediate pro-inflammatory signaling by increasing the expression of NF-κB-activating kinase, IKKi [12]. Conversely, Nur77 may mediate anti-inflammatory signaling by inducing the expression of IκBα, an anti-inflammatory modulator, and subsequently attenuate endothelial cell activation [13]. Recent studies have also shown that Nur77 was protective against the development of atherosclerosis by inhibiting the inflammatory responses [14]. Combined, these data indicate that Nur77 is involved in the regulation of inflammation and its potential functions depend on different physiologic and pathological conditions. However, the *in vivo* functions of Nur77 in inflammation are yet to be substantiated.

In order to further clarify the *in vivo* function of Nur77 in inflammation, we analyzed the phenotype and pathological features of Nur77^−/−^ mice. Our *in vivo* investigations showed that Nur77 deficiency in mice increased their susceptibility to systemic inflammation, indicating that Nur77 participates in the pathogenesis of inflammation.

## Materials and methods

### Mice

Nur77^+/+^ and Nur77^−/−^ mice were obtained from the Jackson Laboratory (Bar Harbor, Maine, USA). All mice were maintained in a pathogen-free environment with a 12-h/12-h light–dark cycle at the Laboratory Animal Center in Soochow University (China), and were provided with normal laboratory pellet diet and water. Animal chow was sterilized by irradiation with 60Co-γ rays. All procedures were performed in accordance with the guidelines of the Animal Care and Use Committee of Soochow University.

### Isolation and culture of peritoneal macrophages

Peritoneal macrophages were isolated from Nur77^+/+^ and Nur77^−/−^ mice. Mice were intraperitoneally (i.p) injected with 2 ml of 4 % thioglycolate medium (BD Biosciences) for 3d, then, macrophages in the peritoneal exudates were collected by peritoneal lavage with 10 ml ice-cold DMEM. Collected cells were incubated in DMEM supplemented with 10 % fetal bovine serum (FBS) at 37 °C for 4 h and washed with PBS to eliminate non-adherent cells. The adherent cells were taken as peritoneal macrophages.

### Tissue samples collection and evaluation

The organs including kidney, liver, lung and spleen from Nur77^+/+^ and Nur77^−/−^ mice were collected for pathological analysis. The spleen weight was measured and all tissues were fixed in 10 % neutral-buffered formalin and embedded in paraffin. After sectioning, the tissues were stained with hematoxylin and eosin (H&E) according to standard histological procedures.

### qPCR Analysis

qPCR analysis was performed as previously described [5]. β-actin was used as internal control. The primers for PCR reactions are listed in Table 1.

**Table 1:** Primers for qPCR

Mouse gene name	Forward/Reverse
*Tnfα*	F: 5′-CTCACACTCAGATCATCTTCTC-3′
	R: 5′-CTTTCTCCTGGTATGAGATAGC-3′
*Il6*	F: 5′-TTCCATCCAGTTGCCTTCTTG-3′
	R: 5′-AGGTCTGTTGGGAGTGGTATC-3′
*Cxcl11*	F: 5′-AGGAAGGTCACAGCCATAGC-3′
	R: 5′-CGATCTCTGCCATTTTGACG-3′
*Indo*	F: 5′-CACTGAGCACGGACGGACTGAGA-3′
	R: 5′-TCCAATGCTTTCAGGTCTTGACGC-3′
*Mrc1*	F: 5′-AGAGCCCACAACAACTCCTG-3′
	R: 5′-TCCACTGCTCGTAATCAGCC-3′
*β-actin*	F: 5′-TGGAATCCTGTGGCATCCATGAAAC-3′
	R: 5′-TAAAACGCAGCTCAGTAACAGTCCG-3′

### ELISA assay

The concentrations of interleukin (IL)-6, immunoglobulin (Ig)G1, and IgE in serum were determined using commercially available kits (eBioscience) according to the manufacturer’s instructions.

### Statistical analysis

Data were expressed as mean ± SD. Each assay was performed in three independent experiments. Statistics was analyzed using Student’s *t* test. Values of *p* < 0.05 were considered statistically significant.

## Results and discussion

**Fig. 1:**
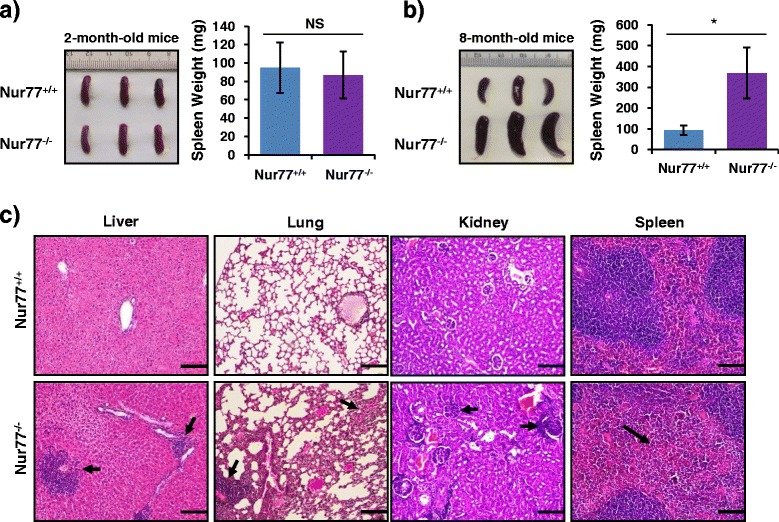
Nur77-deficient mice are more prone to develop systemic inflammation. (**a** and **b**) Gross appearances (left) and weight (right) of spleens from 2-month-old (**a**) and 8-month-old (**b**) Nur77^+/+^ and Nur77^−/−^ mice (*n* = 6 per group). Representative images are shown. NS, not significant, *p < 0.05. (**c**) Hematoxylin and eosin (H&E) staining of indicated organ sections from 8-month-old Nur77^+/+^ and Nur77^−/−^ mice. Representative images are shown. Scale bars, 100 μM. Original magnification, ×100. Arrowheads indicate exacerbation of inflammatory pathology

Recent studies suggest that Nur77 deficiency in mice enhances atherosclerosis [3, 4] and LPS-induced sepsis [15], suggesting that Nur77 is implicated in inflammation. To further evaluate the potential role of Nur77 in inflammation *in vivo*, Nur77^−/−^ mice were observed in this study. We compared splenic differences in 2-month-old Nur77^−/−^ mice and wild-type (WT) mice and found no significant differences in the size and weight of their spleens (Fig. 1a). Interestingly, we found that 8-month-old Nur77^−/−^ mice were more prone to develop splenomegaly (Fig. 1b, left) and increased spleen weight (Fig. 1b, right). Thus, these observations indicated that Nur77 deficiency was associated with an increased inflammatory pathology in elderly mice. Our results were consistent with a recent report that Nur77^−/−^ mice displayed hyperplasia in the jejunum and colon, and increased crypt depth and villus length of the jejunum and colon [16].

Our investigation further showed that Nur77 deficiency in elderly mice led to exacerbation of inflammatory pathology as characterized by increased inflammatory cell infiltration and inflammatory cytokines production. As shown in Fig. 1c, Nur77^−/−^ mice had more severe inflammatory cell infiltration in the liver, lung, spleen and kidney, and more hyperplasia of fibrous tissue in the lungs. The elderly Nur77-deficient mice also exhibited increased susceptibility to glomerulonephritis with enlargement of the kidney glomeruli, which can be linked to systemic disorders or certain infections [17, 18]. Additionally, the spleens of elderly Nur77^−/−^ mice showed expansion of red pulp and decreased white pulp than WT mice (Fig. 1c). Combined, these phenotypical findings suggest a progression of systemic inflammation in Nur77^−/−^ elderly mice.

**Fig. 2:**
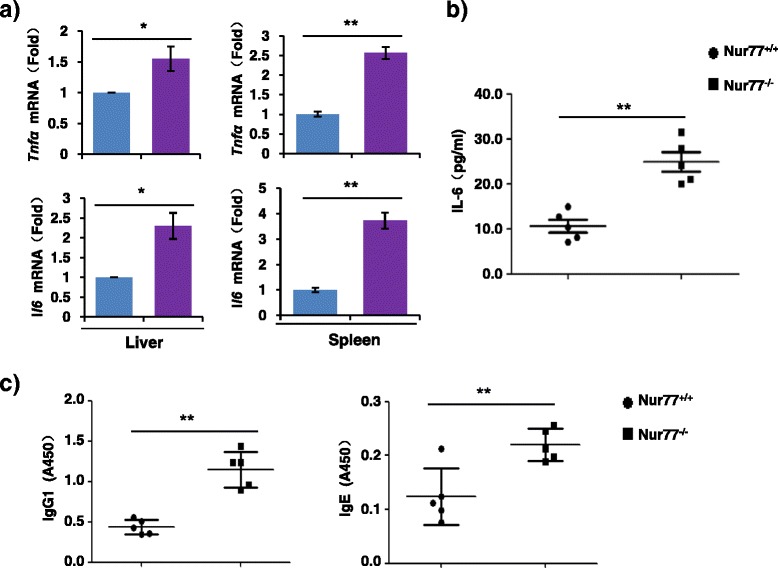
Nur77 deficiency in mice enhances the production of pro-inflammatory cytokines and immunoglobulin. (**a**) qRT-PCR analysis of the expression of *Tnfα* and *Il6* mRNA in liver and spleen samples from 8-monthold Nur77^+/+^ and Nur77^−/−^ mice (n = 6 per group). Error bars represent mean ± s.d. from n = 3 biological triplicates. *p < 0.05 and **p < 0.01. (**b**) ELISA assay of IL-6 concentration in serum from 8-month-old Nur77^+/+^ and Nur77^−/−^ mice (n = 6 per group). Error bars represent mean ± s.d. from n = 3 biological triplicates. **p < 0.01. (**c**) Titers of IgG1 and IgE in serum from 8-month-old Nur77^+/+^ and Nur77^−/−^ mice (n = 6 per group). Error bars represent mean ± s.d. from n = 3 biological triplicates. **p < 0.01

Changes in inflammatory cytokines expression are known to readily occur in systemic inflammation [19]. Unsurprisingly, the mRNA expression of pro-inflammatory cytokines, including *Tnfα* and *Il6*, was higher in the liver and spleen tissues (Fig. 2a) from Nur77^−/−^ mice than those from Nur77^+/+^ mice. The concentration of IL-6 in serum of 8-month-old Nur77^−/−^ mice was also elevated (Fig. 2b). There were no differences in the gene expression of *Tnfα* and *Il6* in 2-month-old mice in the two groups (Additional file 1: Figure S1a). A recent study found that mice lacking all Nr4a receptors including Nur77 had impaired regulatory T cell (Treg) development and resulted in systemic lethal autoimmunity [20], suggesting a potential role of Nr4a receptors in autoimmune disease. Nur77 has also been suggested to be implicated in Treg differentiation and self-reactive T cells selection [21, 22]. Herein, we also investigated whether Nur77 deficiency in mice was involved in the pathogenesis of autoimmune disease. Analysis of serum immunoglobulin showed increased IgG1 and IgE in elderly Nur77^−/−^ mice than in WT mice (Fig. 2c). However, we did not observe any differences in the Ig levels in younger 2-month-old mice in the two groups (Additional file 1: Figure S1b). Together, these data indicate that elderly Nur77^−/−^ mice developed systemic inflammation and autoimmunity, supporting a role of Nur77 in inflammation and immunity. Our results are consistent with previous reports that Nur77^−/−^ mice showed increased susceptibility to atherosclerosis [4, 14] and restenosis [10], and demonstrate that Nur77 has functional roles in protecting mice from inflammatory and autoimmune diseases.

**Fig. 3:**
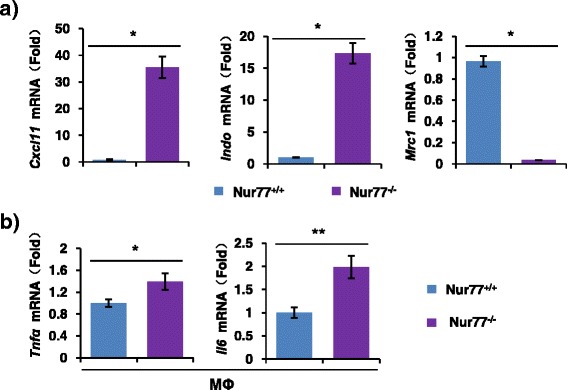
Nur77 deletion polarizes macrophages toward a pro-inflammatory M1 phenotype. (**a**) The expression of *Cxcl11*, *Indo*, and *Mrc1* were determined by qRT-PCR in peritoneal macrophages (MΦ) of 8-month-old Nur77^+/+^ (*n* = 6) and Nur77^−/−^ (*n* = 6) mice. Error bars represent mean ± s.d. from *n* = 3 biological triplicates. *p < 0.05. (**b**) The expression of *Tnfα* and *Il6* mRNA were analyzed by qRT-PCR in peritoneal macrophages (MΦ) from 8-month-old Nur77^+/+^ (*n* = 6) and Nur77^−/−^ (*n* = 6) mice. Error bars represent mean ± s.d. from *n* = 3 biological triplicates. *p < 0.05 and **p < 0.01

Accumulating studies have shown that polarization of macrophages occurs in response to inflammatory diseases [23]. Two opposite and competing macrophage phenotypes, M1 (pro-inflammatory) and M2 (anti-inflammatory), have been defined and identified in a range of physiologic and pathological processes [23]. Several genes, including CXCL11, INDO, and MRC1, have been shown to discriminate between M1 and M2 macrophages [24]. M1 phenotype is typically associated with an increased CXCL11 and INDO expression but associated with a decreased MRC1 expression [24]. In this study, we isolated peritoneal macrophages from elderly 8-month-old Nur77^+/+^ and Nur77^−/−^ mice, and found that Nur77 deficiency in mice significantly enhanced *Cxcl11* and *Indo* mRNA expression, but reduced *Mrc1* expression (Fig. 3a), indicating that the absence of Nur77 in macrophages led to enhanced polarization of macrophages toward a pro-inflammatory M1 phenotype. M1-like macrophages are also generally considered to produce numerous pro-inflammatory mediators such as TNFα, IL-12, and IL-6 [25]. Similar phenomena were also observed in our current study. As shown in Fig. 3b, Nur77 deficiency in peritoneal macrophages markedly enhanced the expression of *Tnfα* and *Il6*, indicating that Nur77 plays an important role in regulation of macrophage phenotype and functions. These results are strongly supported by a recent report that Nur77 deletion polarizes macrophages toward an inflammatory phenotype and increases inflammatory cytokines production in atherosclerosis [14]. Together with our observations that increased inflammatory cells infiltration and inflammatory cytokines production in elderly Nur77^−/−^ mice (Figs. 1 and 2), thus, we speculate that Nur77-deficiency skews the macrophage phenotype to M1 subset, and subsequently leads to the development of systemic inflammation in elderly mice.

In summary, we provide *in vivo* evidence that orphan nuclear receptor Nur77 is an essential negative regulator of systemic inflammation. This study associates the nuclear receptor Nur77 with systemic inflammation and implicates a new therapeutic target to preventing and treating inflammatory diseases.

## Additional file

Additional file 1:
**Figure S1.** Nur77 deficiency in 2-month-old mice does not affect pro-inflammatory cytokines expression and immunoglobulin production. (a) The expression of *Tnfα* and *Il6* mRNA were analyzed by qRT-PCR in liver and spleen samples from 2-month-old Nur77^+/+^ and Nur77^−/−^ mice (*n* = 6 per group). Error bars represent mean ± s.d. from *n* = 3 biological triplicates. (b) Titers of IgG1 and IgE in serum from 2-month-old Nur77^+/+^ and Nur77^−/−^ mice (*n* = 6 per group). Error bars represent mean ± s.d. from *n* = 3 biological triplicates.

## Abbreviations

qPCR: Quantitative PCR
ELISA: Enzyme Linked Immunosorbent Assay
CXCL11: Chemokine (C-X-C motif) ligand 1
IDO1: Indoleamine 2,3-dioxygenase 1
MRC1: Mannose receptor, C type 1

## References

[1] Lee SO, Li X, Khan S, Safe S (2011). Targeting NR4A1 (TR3) in cancer cells and tumors. Expert Opin Ther Targets.

[2] Zhan YY, Chen Y, Zhang Q, Zhuang JJ, Tian M, Chen HZ, Zhang LR, Zhang HK, He JP, Wang WJ (2012). The orphan nuclear receptor Nur77 regulates LKB1 localization and activates AMPK. Nat Chem Biol.

[3] Arkenbout EK, de Waard V, van Bragt M, van Achterberg TA, Grimbergen JM, Pichon B, Pannekoek H, de Vries CJ (2002). Protective function of transcription factor TR3 orphan receptor in atherogenesis: decreased lesion formation in carotid artery ligation model in TR3 transgenic mice. Circulation.

[4] Hamers AA, Vos M, Rassam F, Marinkovic G, Kurakula K, van Gorp PJ, de Winther MP, Gijbels MJ, de Waard V, de Vries CJ (2012). Bone marrow-specific deficiency of nuclear receptor Nur77 enhances atherosclerosis. Circ Res.

[5] Wang JR, Gan WJ, Li XM, Zhao YY, Li Y, Lu XX, Li JM, Wu H (2014). Orphan nuclear receptor Nur77 promotes colorectal cancer invasion and metastasis by regulating MMP-9 and E-cadherin. Carcinogenesis.

[6] Lee SO, Abdelrahim M, Yoon K, Chintharlapalli S, Papineni S, Kim K, Wang H, Safe S (2010). Inactivation of the orphan nuclear receptor TR3/Nur77 inhibits pancreatic cancer cell and tumor growth. Cancer Res.

[7] Bonta PI, van Tiel CM, Vos M, Pols TW, van Thienen JV, Ferreira V, Arkenbout EK, Seppen J, Spek CA, van der Poll T (2006). Nuclear receptors Nur77, Nurr1, and NOR-1 expressed in atherosclerotic lesion macrophages reduce lipid loading and inflammatory responses. Arterioscler Thromb Vasc Biol.

[8] Achiron A, Feldman A, Gurevich M (2011). Characterization of multiple sclerosis traits: nuclear receptors (NR) impaired apoptosis pathway and the role of 1-alpha 25-dihydroxyvitamin D3. J Neurol Sci.

[9] Pei L, Castrillo A, Chen M, Hoffmann A, Tontonoz P (2005). Induction of NR4A orphan nuclear receptor expression in macrophages in response to inflammatory stimuli. J Biol Chem.

[10] Papac-Milicevic N, Breuss JM, Zaujec J, Ryban L, Plyushch T, Wagner GA, Fenzl S, Dremsek P, Cabaravdic M, Steiner M (2012). The interferon stimulated gene 12 inactivates vasculoprotective functions of NR4A nuclear receptors. Circ Res.

[11] Uhrin P, Perkmann T, Binder B, Schabbauer G (2013). ISG12 is a critical modulator of innate immune responses in murine models of sepsis. Immunobiology.

[12] Pei L, Castrillo A, Tontonoz P (2006). Regulation of macrophage inflammatory gene expression by the orphan nuclear receptor Nur77. Mol Endocrinol.

[13] You B, Jiang YY, Chen S, Yan G, Sun J (2009). The orphan nuclear receptor Nur77 suppresses endothelial cell activation through induction of IkappaBalpha expression. Circ Res.

[14] Hanna RN, Shaked I, Hubbeling HG, Punt JA, Wu R, Herrley E, Zaugg C, Pei H, Geissmann F, Ley K, Hedrick CC (2012). NR4A1 (Nur77) deletion polarizes macrophages toward an inflammatory phenotype and increases atherosclerosis. Circ Res.

[15] Li L, Liu Y, Chen HZ, Li FW, Wu JF, Zhang HK, He JP, Xing YZ, Chen Y, Wang WJ (2015). Impeding the interaction between Nur77 and p38 reduces LPS-induced inflammation. Nat Chem Biol.

[16] Chen HZ, Liu QF, Li L, Wang WJ, Yao LM, Yang M, Liu B, Chen W, Zhan YY, Zhang MQ (2012). The orphan receptor TR3 suppresses intestinal tumorigenesis in mice by downregulating Wnt signalling. Gut.

[17] Zand L, Fervenza FC, Nasr SH, Sethi S (2014). Membranoproliferative glomerulonephritis associated with autoimmune diseases. J Nephrol.

[18] Couser WG, Johnson RJ (2014). The etiology of glomerulonephritis: roles of infection and autoimmunity. Kidney Int.

[19] Aksentijevich I. Update on genetics and pathogenesis of autoinflammatory diseases: the last 2 years. Semin Immunopathol. 2015. [Epub ahead of print]. 10.1007/s00281-015-0478-4 25860799

[20] Sekiya T, Kashiwagi I, Yoshida R, Fukaya T, Morita R, Kimura A, Ichinose H, Metzger D, Chambon P, Yoshimura A (2013). Nr4a receptors are essential for thymic regulatory T cell development and immune homeostasis. Nat Immunol.

[21] Fassett MS, Jiang W, D’Alise AM, Mathis D, Benoist C (2012). Nuclear receptor Nr4a1 modulates both regulatory T-cell (Treg) differentiation and clonal deletion. Proc Natl Acad Sci U S A.

[22] Zhou T, Cheng J, Yang P, Wang Z, Liu C, Su X, Bluethmann H, Mountz JD (1996). Inhibition of Nur77/Nurr1 leads to inefficient clonal deletion of self-reactive T cells. J Exp Med.

[23] Labonte AC, Tosello-Trampont AC, Hahn YS (2014). The role of macrophage polarization in infectious and inflammatory diseases. Mol Cells.

[24] Stossi F, Madak-Erdogan Z, Katzenellenbogen BS (2012). Macrophage-elicited loss of estrogen receptor-alpha in breast cancer cells via involvement of MAPK and c-Jun at the ESR1 genomic locus. Oncogene.

[25] Liu YC, Zou XB, Chai YF, Yao YM (2014). Macrophage polarization in inflammatory diseases. Int J Biol Sci.

