# Supplementary figures and images for: Nur77 deficiency leads to systemic inflammation in elderly mice
Source: J Inflamm (Lond). 2015 Jun 26;12:40. doi: 10.1186/s12950-015-0085-0 (PMC4480882; doi:10.1186/s12950-015-0085-0)

# Figure S1

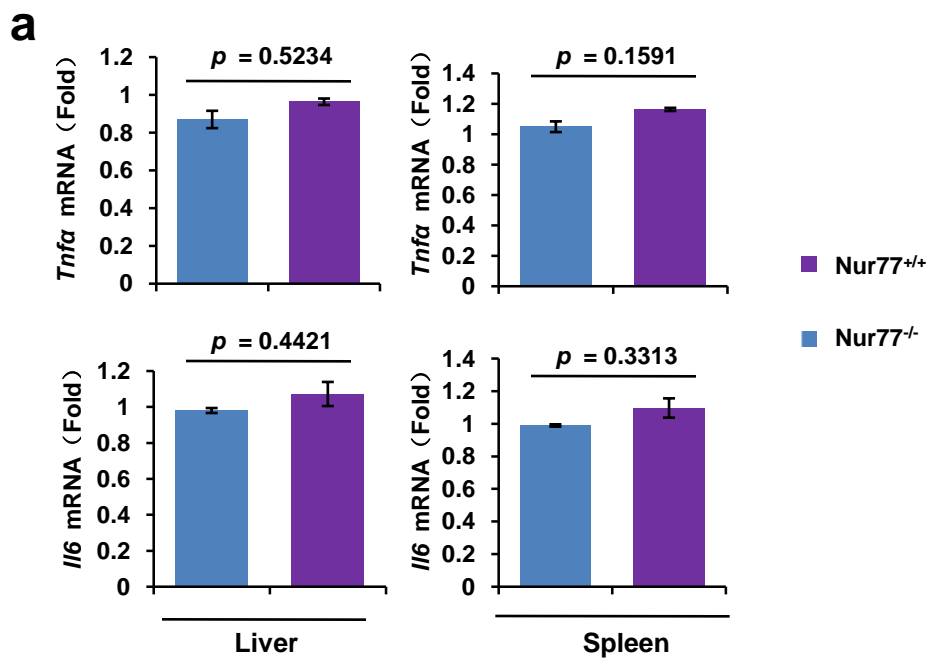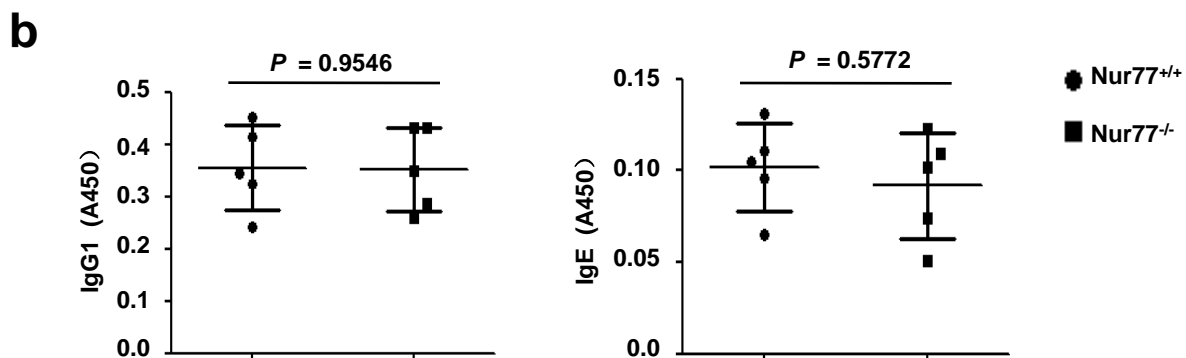

Supplement: Additional file 1: — Figure S1. Nur77 deficiency in 2-month-old mice does not affect pro-inflammatory cytokines expression and immunoglobulin production. (a) The expression of Tnfα and Il6 mRNA were analyzed by qRT-PCR in liver and spleen samples from 2-month-old Nur77+/+ and Nur77−/− mice (n = 6 per group). Error bars represent mean ± s.d. from n = 3 biological triplicates. (b) Titers of IgG1 and IgE in serum from 2-month-old Nur77+/+ and Nur77−/− mice (n = 6 per group). Error bars represent mean ± s.d. from n = 3 biological triplicates. [file 12950_2015_85_MOESM1_ESM.pdf]
